# Implementation of an institutional Enhanced Recovery After Surgery (ERAS) pathway is associated with fewer in‐hospital falls after total knee arthroplasty

**DOI:** 10.1002/jeo2.70895

**Published:** 2026-08-26

**Authors:** Judith Ewert, Yizhou Ge, Martin Häner, Jan‐Peter Braun, Wolf Petersen

**Affiliations:** ^1^ Department of Orthopedics Martin Luther Hospital Berlin Germany; ^2^ Charité—Universitätsmedizin Berlin, Freie Universität Berlin and Humboldt‐Universität zu Berlin Berlin Germany; ^3^ Department of Anaesthesiology Martin Luther Hospital Berlin Germany

**Keywords:** fall prevention, fast track surgery, knee replacement, knee surgery, osteoarthritis

## Abstract

**Purpose:**

To evaluate whether implementation of an Enhanced Recovery After Surgery (ERAS)‐based perioperative pathway was associated with a lower rate of postoperative in‐hospital falls after total knee arthroplasty (TKA).

**Methods:**

This retrospective single‐centre before‐after study included 314 patients undergoing primary TKA (151 treated before and 163 treated after implementation of an ERAS‐based pathway). The documented pathway components included local infiltration analgesia, tranexamic acid, omission of surgical drains and continuous femoral nerve analgesia and physiotherapist‐assisted active and passive mobilization on the day of surgery. The primary outcome was an in‐hospital fall documented through the institutional incident‐reporting system. Secondary outcomes included pain, opioid consumption, range of motion and postoperative complications.

**Results:**

In‐hospital falls occurred in 1 of 163 patients in the ERAS group (0.6%) and 10 of 151 patients in the non‐ERAS group (6.6%) (risk ratio, 0.09; 95% confidence interval [CI], 0.01–0.72; *p* = 0.004), corresponding to an absolute risk reduction of 6.0 percentage points (95% CI, 1.9–11.2 percentage points). Three falls required medical treatment. Pain at rest was comparable, whereas opioid consumption was lower in the ERAS group (*p* < 0.001). Time to achieve 90° of knee flexion was shorter in the ERAS group (*p* < 0.001). Postoperative complications occurred in 0 of 163 ERAS patients and 5 of 151 non‐ERAS patients (0% vs. 3.3%; *p* = 0.025).

**Conclusions:**

Implementation of an ERAS‐based perioperative pathway was associated with fewer postoperative in‐hospital falls after primary TKA. Because the pathway was introduced as a bundle in a retrospective before‐after study, causality cannot be established.

**Level of Evidence:**

Level III, retrospective cohort study.

**Abbreviations**
ASA: American Society of Anesthesiologists
BMI: body mass index
CPM: continuous passive motion
DVT: deep vein thrombosis
ERAS: Enhanced Recovery After Surgery
LIA: local infiltration analgesia
TKA: total knee arthroplasty
VAS: visual analogue scale

## INTRODUCTION

Total knee arthroplasty (TKA) is an established treatment for end‐stage knee osteoarthritis, providing substantial pain relief and functional improvement [1, 7]. Nevertheless, in‐hospital falls remain a clinically relevant perioperative complication because they may cause injury, delay rehabilitation, prolong hospital stay and increase healthcare utilization [2, 8, 12]. Although patients undergoing elective TKA are not traditionally considered a high‐risk population, inpatient falls are not negligible. Wasserstein et al. reported an in‐hospital fall rate of 2.7% in a registry‐based cohort of 2197 patients following primary TKA [15].

The risk of falling during the early postoperative period is likely multifactorial. Patient‐related factors include advanced age, obesity, comorbidities, frailty and impaired preoperative mobility, whereas perioperative contributors include postoperative pain, opioid‐related sedation or dizziness, orthostatic intolerance, impaired proprioception and quadriceps weakness [2, 8, 12, 15]. Continuous femoral nerve block has received particular attention because motor blockade may compromise quadriceps function and early postoperative stability [2, 14, 15]. Accordingly, both analgesic strategy and the timing and supervision of mobilization should be considered when evaluating fall risk after TKA.

Enhanced Recovery After Surgery (ERAS) pathways combine several perioperative interventions intended to accelerate functional recovery and reduce treatment‐related morbidity. In TKA, relevant components include motor‐sparing analgesia, reduced opioid exposure, blood management, omission of unnecessary drains and early physiotherapist‐supervised mobilization [5, 6, 11, 13, 18, 19]. Local infiltration analgesia (LIA) provides pain control comparable to femoral nerve block while preserving quadriceps motor function [9, 10, 16, 17]. However, previous meta‐analyses have not demonstrated a consistent reduction in falls.

Although ERAS pathways have been associated with faster recovery and shorter hospital stay after TKA [3, 4, 5, 6, 18, 19], their relationship with in‐hospital falls remains insufficiently investigated. The purpose of the present study was therefore to evaluate whether implementation of an ERAS‐based perioperative pathway was associated with a lower prevalence of in‐hospital falls after primary TKA. We hypothesized that patients treated within the ERAS‐based pathway would experience fewer in‐hospital falls than patients managed using the preceding conventional pathway.

## METHODS

### Ethics approval and informed consent

The study was approved by the Ethics Committee of the Faculty of Medicine at Charité—Universitätsmedizin Berlin (approval number: EA2/137/20). All patients provided informed consent for collection of postoperative patient‐reported outcomes and scientific use of data recorded during the inpatient stay.

### Patients

This retrospective single‐centre before‐after study compared outcomes before and after implementation of an ERAS‐based perioperative pathway. All procedures were performed at one institution by the same two senior surgeons using the same implant system, alignment philosophy and standardized surgical technique. Nevertheless, changes over time unrelated to the pathway could not be excluded.

Group A (non‐ERAS) included all patients who underwent primary TKA between 1 January 2017 and 31 December 2017, before implementation of the ERAS‐based perioperative pathway.

The period between 1 January 2018 and 31 March 2018 represented the transition phase during implementation of the ERAS pathway. Patients treated during this period who received a combination of femoral nerve block and LIA were excluded from the final analysis to ensure a clear separation between groups.

Group B (ERAS) included all patients who underwent primary TKA between 1 April 2018 and 31 March 2019, after implementation of the ERAS‐based perioperative pathway.

### Perioperative protocols

Patients were treated either according to the conventional perioperative pathway or within an institutional ERAS‐based pathway. The documented differences included analgesic technique, tranexamic acid use, surgical drainage and timing of mobilization. Mobilization included both active and passive exercises; however, the use of a continuous passive motion (CPM) machine was not systematically documented or analysed as a separate intervention.

In contrast, patients in the conventional (non‐ERAS) group were managed according to the standard care pathway in place prior to ERAS implementation, which was characterized by the use of a femoral nerve block for postoperative analgesia, routine use of surgical drains and delayed initiation of postoperative mobilization.

The documented perioperative pathway included preoperative assessment using the American Society of Anesthesiologists (ASA) classification, general or spinal anaesthesia, perioperative antibiotic prophylaxis with cefuroxime and a standardized surgical technique using the same implant system and alignment philosophy in both groups. The principal differences between the treatment periods concerned analgesia, blood management, drainage and timing of mobilization. The conventional pathway included continuous femoral nerve analgesia, routine Redon drainage and physiotherapy beginning on the first postoperative day. The ERAS‐based pathway included LIA, preoperative tranexamic acid, omission of continuous femoral nerve analgesia and surgical drains and physiotherapist‐assisted mobilization on the day of surgery, no earlier than 3 h postoperatively. In both groups, full weight bearing as tolerated using two crutches was permitted and range of motion was unrestricted. Other elements of a comprehensive ERAS Society pathway were not systematically assessed; therefore, the pathway is described as ERAS‐based rather than as a fully compliant ERAS protocol. Table 1 lists the selected ERAS‐related components documented for the pathway evaluated in this study.

**Table 1:** Differences between the conventional and ERAS‐based pathways.

Pathway component	Conventional pathway	ERAS‐based pathway
Mobilization and physiotherapy	Physiotherapy beginning on postoperative Day 1	Physiotherapist‐assisted mobilization on the day of surgery, no earlier than 3 h postoperatively
Tranexamic acid	Not routinely administered	Intravenous administration of 1 g tranexamic acid 30 min before surgery
Analgesic technique	Continuous femoral nerve block	Intra‐articular infiltration analgesia with 150 mL ropivacaine solution at the end of surgery
Surgical drainage	Routine Redon drainage	No Redon drainage
Pain catheter/pump	Continuous pain catheter and pump	No pain catheter or pump

*Note*: Both groups received the same preoperative assessment, anaesthesia options, antibiotic prophylaxis, surgical technique, implant, alignment strategy, weight‐bearing protocol and unrestricted range‐of‐motion regimen. Comprehensive adherence to all ERAS Society recommendations was not assessed.

Abbreviation: ERAS, Enhanced Recovery After Surgery.

**Figure 1:**
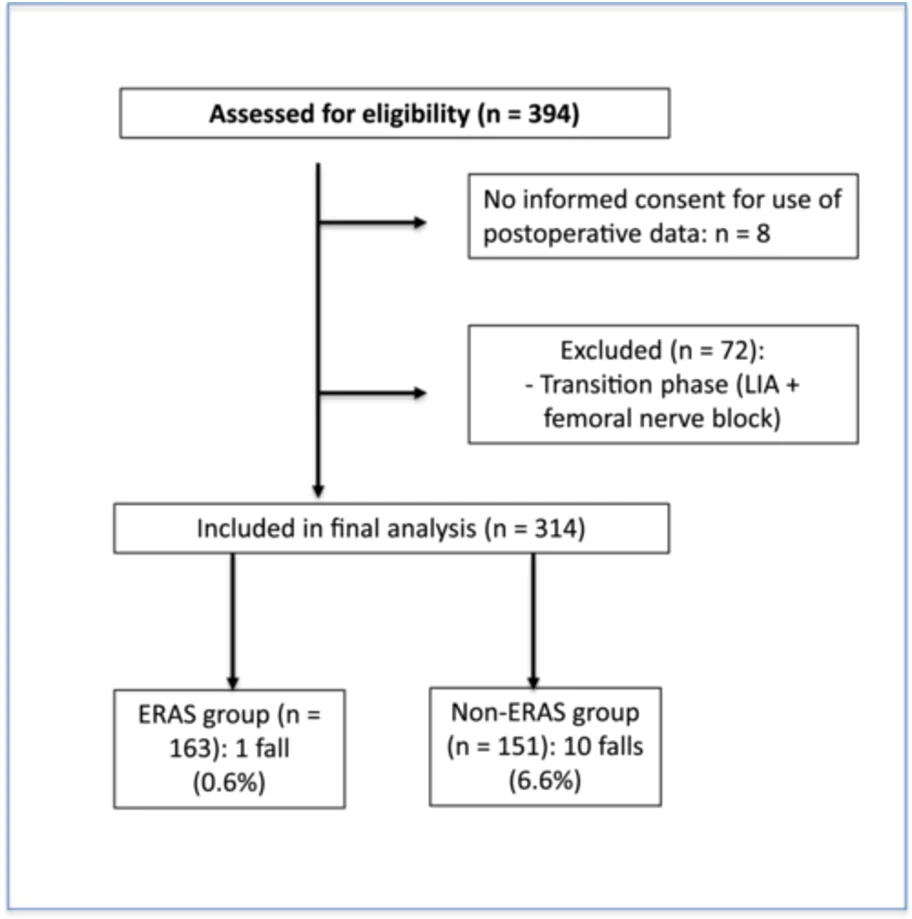
Flowchart of patient inclusion and analysis. ERAS, Enhanced Recovery After Surgery; LIA, local infiltration analgesia.

Inclusion criteria were the implantation of a primary TKA and informed consent that postoperative data (patient‐reported outcome measures) can also be collected, and that the data collected during the inpatient stay may be used for scientific purposes. Exclusion criteria were revision surgery, tibial tubercle osteotomy, bilateral TKA, partial knee arthroplasty or implantation of a rotating hinge prosthesis or the combination of both femoral nerve block and LIA (Table 2). Patients treated during the transition phase with a combination of analgesic protocols (LIA and femoral nerve block) were excluded to ensure a clear separation between study groups (Figure 1).

**Table 2:** Inclusion and exclusion criteria.

Inclusion criteria	Exclusion criteria
– Implantation of a primary TKA	– Revision surgery – Tibial tubercle osteotomy – Bilateral TKA – Partial knee arthroplasty – Rotating hinge prosthesis – Combination of both femoral nerve block and LIA – No informed consent to collect postoperative data for the present study

Abbreviations: LIA, local infiltration analgesia; TKA, total knee arthroplasty.

### Surgical technique

All patients in the non‐ERAS group received a preoperative femoral nerve block with a continuous pain pump. The nerve block was performed by experienced anesthesiologists under ultrasound guidance. After identification of the femoral nerve, a bolus of 0.75% ropivacaine was administered, followed by placement of a continuous pain pump delivering 0.375% ropivacaine at a flow rate of 6 mL/h.

In the ERAS group, all patients received 1 g of tranexamic acid preoperatively.

The surgical technique for TKA was identical in both groups. Procedures were performed under general or spinal anaesthesia. Perioperative antibiotic prophylaxis consisted of cefuroxime (1.5 g).

All TKAs were implanted using a mechanical alignment philosophy with restoration of a neutral mechanical axis and balanced flexion and extension gaps. A standard anteromedial approach with a medial split of the quadriceps tendon and a longitudinal split of the medial retinaculum medial to the patellar tendon was used to expose the joint.

In all patients, a cruciate‐retaining bicondylar knee prosthesis without patellar resurfacing was implanted using a femur‐first technique (Vanguard CR; Zimmer Biomet). The patella was everted during implantation and cementation of the femoral and tibial components.

After cementation, the anteromedial capsular incision was closed with resorbable braided sutures (Vicryl 1) and the skin was closed with staples.

Routine closed‐suction drainage (Redon drainage) was used in all patients of the non‐ERAS group, whereas drains were omitted in all patients treated within the ERAS pathway.

In the ERAS group, LIA was performed intraoperatively before cementation. The posterior capsule, periarticular ligaments, Hoffa's fat pad, subcutaneous tissue and the region of the saphenous nerve at the medial femoral condyle were infiltrated with 150 mL ropivacaine [13].

### Rehabilitation

All patients were mobilized with full weight bearing as tolerated using two crutches. Range of motion was not restricted. Thus, the rehabilitation protocols were identical between groups except for the timing of mobilization.

In the ERAS group, physiotherapy was routinely initiated on the day of surgery, no earlier than 3 h postoperatively. To facilitate implementation of the ERAS pathway, physiotherapy working schedules were adapted to ensure availability on the day of surgery, including for patients undergoing procedures later in the day. Review of the medical records confirmed that all patients in the ERAS group received physiotherapist‐assisted mobilization on the day of surgery.

In the non‐ERAS group, physiotherapy started on the first postoperative day.

### General data analysis

Descriptive patient data were collected and compiled in an Excel database, including sex, age, ASA classification and medication.

The ASA classification was used to assess the patients' preoperative health status according to the ASA I–VI classification. ASA grading was performed by the anesthesiologist as part of the routine preoperative assessment.

### Primary endpoint

The primary endpoint of this study was the occurrence of in‐hospital falls. A fall was defined as any unintentional descent to the floor or a lower level occurring during the in‐hospital stay, irrespective of injury severity. All falls were prospectively documented using a standardized reporting form as part of our institutional patient safety and incident reporting system, which requires mandatory reporting by the clinical staff. The standardized fall‐report protocol includes documenting the time of the fall, circumstances of the fall, mechanism of the fall and resulting injuries.

### Secondary endpoints

Secondary endpoints included pain intensity, opioid consumption, range of motion and the occurrence of complications (e.g., thromboembolic or cardiopulmonary events).

Pain intensity was assessed using a visual analogue scale (VAS) ranging from 0 to 10, where 0 indicated no pain and 10 indicated unbearable pain. Pain levels were routinely documented by the nursing staff during hospitalization and were recorded separately for pain at rest. Data were collected for the day of surgery and the first four postoperative days.

Range of motion was measured daily by physiotherapists using a goniometer during the hospital stay.

Postoperative haemoglobin levels were recorded as part of routine clinical care, with a standard assessment performed preoperatively and on the second postoperative day. Haemoglobin decline was defined as the difference between the preoperative value and the lowest postoperative value measured during the in‐hospital stay. These data were analysed to assess potential differences in perioperative blood loss between the ERAS and non‐ERAS groups.

Opioid consumption and complications were extracted from the electronic medical records. Opioid doses were converted into morphine equivalent doses (mg) using the Opioid Rotation Tool of the German Pain Society (Deutsche Schmerzgesellschaft e.V.).

### Statistical methods

All collected patient parameters were compiled in an Excel database (Microsoft Excel; Microsoft Corporation). The dataset was subsequently pseudonymized. Identifiable data were accessible only to the principal investigator and an authorized study collaborator.

Continuous variables were compared using the Mann–Whitney *U* test. Categorical variables, including the primary outcome of in‐hospital falls, were compared using Fisher's exact test because of the small number of events. Binary outcomes were additionally reported with effect estimates and corresponding 95% confidence intervals. Differences with *p* < 0.05 were considered statistically significant.

Statistical analyses and data visualization were performed using R software (Version 4.1; R Foundation for Statistical Computing).

## RESULTS

### Patient characteristics

A total of 394 patients undergoing primary TKA between 2017 and 2019 were screened. Eighty were excluded: 72 received both femoral nerve block and LIA during the transition period, and 8 had not consented to scientific use of postoperative data. The final analysis included 314 patients (151 non‐ERAS and 163 ERAS). Medical‐record review confirmed physiotherapist‐assisted mobilization on the day of surgery for all patients in the ERAS group.

The distribution of anaesthesia type was comparable between groups, with general anaesthesia used in 76.7% of patients in the ERAS group and 77.7% of patients in the non‐ERAS group (*p* = 0.85). Patient characteristics are summarized in Table 3.

**Table 3:** Characteristics of patients in the two treatment groups.

	ERAS group *N* = 163	Non‐ERAS group *N* = 151	*p* value
Age, median [range]	73 [50−89]	72 [46−89]	0.219
Gender, *N* (%)	Male: 69 (42.3%) Female: 94 (57.7%)	Male: 58 (38.7%) Female: 93 (61.3%)	0.915
BMI, mean ± SD	30.8 ± 2.7	30.0 ± 2.1	0.565
ASA grade, median [range]	2.0 [1−3]	2.0 [1−3]	0.327
Preoperative use of anticoagulation	Aspirin: 32 (19.6%) NOACs: 18 (11%) Clopidogrel: 1 (0.6%) Phenprocoumon: 7 (4.3%) None: 105 (64.4%)	Aspirin: 31 (19.5%) NOACs: 10 (6.3%) Clopidogrel: 1 (0.6%) Phenprocoumon: 9 (5.7%) None: 107 (70.9%)	0.633
General anaesthesia	125 (76.7%)	117 (77.7%)	0.85
Spinal anaesthesia	38 (23.3%)	34 (22.3)	

*Note*: Patients could receive more than one anticoagulant medication; therefore, the medication categories are not mutually exclusive, and the sum of categories may exceed the total number of patients.

Abbreviations: ASA, American Society of Anesthesiologists; BMI, body mass index; ERAS, Enhanced Recovery After Surgery; NOACs, Non‐Vitamin K Antagonist Oral Anticoagulants; SD, standard deviation.

### Primary outcome measure: Frequency of in‐hospital falls

In‐hospital falls occurred in 1 of 163 patients in the ERAS group (0.6%) and 10 of 151 patients in the non‐ERAS group (6.6%) (Fisher's exact *p* = 0.004). This corresponded to a risk ratio of 0.09 (95% confidence interval [CI], 0.01–0.72) and an absolute risk reduction of 6.0 percentage points (95% CI, 1.9–11.2 percentage points).

Most patients sustained minor injuries such as bruises and superficial skin abrasions (one patient in the ERAS group and seven in the non‐ERAS group).

A total of three falls were attributed to vasovagal syncope, while eight falls were related to loss of balance.

Falls occurred predominantly within the first postoperative days. One fall was recorded on the day of surgery (Day 0), followed by a peak incidence on postoperative Day 1 with three events. Thereafter, the number of falls decreased, with two falls each on Days 2 and 3, and one fall per day from postoperative Days 4–6. No falls were observed beyond postoperative day 6.

Three patients in the non‐ERAS group experienced falls that required medical treatment. Two of these cases necessitated surgical intervention: One patient underwent skin suturing for a head laceration, and another required revision surgery due to wound rupture with dehiscence. The third patient underwent clinical and radiographic evaluation to exclude fracture and was treated conservatively.

### Secondary outcome measures: Pain and opioid use, range of motion, haemoglobin and other complications

#### Pain and morphine equivalent dose

Pain at rest did not differ significantly between the ERAS and non‐ERAS groups at any time point from the day of surgery to postoperative Day 4 (Table 4). Mean VAS pain scores at rest remained low in both groups throughout the observation period, ranging from 1.66 to 2.73 cm in the ERAS group and from 1.47 to 2.53 cm in the non‐ERAS group. No statistically significant differences were observed between groups on any postoperative day (all *p* > 0.05).

**Table 4:** Comparison of pain at rest measured with the VAS during the first 4 postoperative days between the ERAS and non‐ERAS groups.

Day	ERAS mean SD	Non‐ERAS mean SD	*p* value
0	1.72 ± 1.76 cm	1.47 ± 2.02 cm	0.053
1	2.73 ± 1.86 cm	2.53 ± 2.23 cm	0.088
2	2.18 ± 1.60 cm	2.36 ± 1.86 cm	0.488
3	1.73 ± 1.43 cm	1.63 ± 1.50 cm	0.207
4	1.66 ± 1.41 cm	1.97 ± 1.70 cm	0.153

*Note*: Comparison between the ERAS and non‐ERAS groups using the Mann–Whitney *U* test.

Abbreviations: ERAS, Enhanced Recovery After Surgery; SD, standard deviation; VAS, visual analogue scale.

Similarly, no significant differences in walking pain were observed between the ERAS and non‐ERAS groups during the first four postoperative days (Table 5). Mean VAS scores during walking ranged from 2.54 to 3.85 cm in the ERAS group and from 2.66 to 3.70 cm in the non‐ERAS group (all *p* > 0.05). However, comparison at the day of surgery should be interpreted with caution, as walking pain data at Day 0 were available for only a limited number of patients in the non‐ERAS group (*n* = 10), reflecting the delayed mobilization strategy used in this cohort.

**Table 5:** Comparison of walking pain measured with the VAS during the first 4 postoperative days between the ERAS and non‐ERAS groups.

Day	ERAS mean SD	Non‐ERAS mean SD	*p* value
0	2.54 ± 1.95 cm	2.66 ± 1.78 cm	0.573
1	3.85 ± 1.86 cm	3.68 ± 2.06 cm	0.769
2	3.57 ± 1.58 cm	3.70 ± 1.71 cm	0.557
3	3.04 ± 1.55 cm	3.30 ± 1.46 cm	0.539
4	2.82 ± 1.59 cm	3.08 ± 1.52 cm	0.559

*Note*: Comparison between the ERAS and non‐ERAS groups using the Mann–Whitney *U* test.

Abbreviations: ERAS, Enhanced Recovery After Surgery; SD, standard deviation; VAS, visual analogue scale.

Despite comparable pain levels, opioid consumption was significantly higher in the non‐ERAS group compared with the ERAS group (*p* < 0.001). The mean morphine equivalent dose was 24.5 mg in the non‐ERAS group and 10.2 mg in the ERAS group. The median dose was 20 and 10 mg, respectively.

### Postoperative range of motion

Maximum knee flexion on postoperative Day 4 was significantly greater in the ERAS group compared with the non‐ERAS group (*p* < 0.001), with mean values of 76.7° ± 16.07 and 67.4° ± 18.65, respectively. No significant difference was observed between the groups regarding knee extension (ERAS: 0.39° ± 1.71° vs. non‐ERAS: 0.47° ± 2.13°).

### Haemoglobin

No significant difference in postoperative haemoglobin decline was observed between the ERAS and non‐ERAS groups. The mean decrease in haemoglobin was 2.75 ± 1.23 g/dL in the ERAS group and 2.72 ± 1.56 g/dL in the non‐ERAS group (*p* = 0.071). Median values were comparable between groups, with 2.8 g/dL (range: 1.3–10.6) in the ERAS group and 2.5 g/dL (range: 0.4–14.0) in the non‐ERAS group.

### Length of hospital stay

The mean length of hospital stay was 7.4 days in the ERAS group and 9.3 days in the non‐ERAS group, demonstrating a statistically significant reduction following ERAS implementation (*p* < 0.001).

### Other complications

No patient in the ERAS group experienced a recorded postoperative complication, whereas five patients in the non‐ERAS group experienced at least one complication (0 of 163 [0%] vs. 5 of 151 [3.3%]; *p* = 0.025). The affected patients and recorded diagnoses are summarized in Table 6; three patients had thromboembolic complications.

**Table 6:** Overview of patients who developed complications.

Group	Complication	Day	Treatment
Non‐ERAS	Vasospastic angina pectoris, sinus tachycardia, Anaemia (Hb 6.8 g/dL)	1	Transfusion of two units of packed red blood cells
Non‐ERAS	Occlusion of the left superficial femoral artery and left popliteal artery (stent)	3	Unsuccessful thrombectomy, emergency bypass (P3), fasciotomy
Non‐ERAS	Deep vein thrombosis (DVT), thrombosis of the popliteal artery (4 cm), small segmental pulmonary embolism	3	Therapeutic dalteparin
Non‐ERAS	Early postoperative infection → revision surgery Fibular vein thrombosis with pulmonary embolism	5	Revision surgery, anticoagulation
Non‐ERAS	Postoperative ileus, cardiac decompensation	10	Adhesiolysis, pharmacological stimulation, resuscitation unsuccessful

Abbreviations: ERAS, Enhanced Recovery After Surgery; Hb, haemoglobin.

## DISCUSSION

The principal finding of the present study was that implementation of an ERAS‐based perioperative pathway was associated with a lower rate of in‐hospital falls after primary TKA. Falls occurred in 0.6% of patients in the ERAS group and 6.6% in the conventional‐care group (risk ratio, 0.09; 95% CI, 0.01–0.72; *p* = 0.004), corresponding to an absolute risk reduction of 6.0 percentage points (95% CI, 1.9–11.2 percentage points). Importantly, this association was observed despite earlier physiotherapist‐supervised mobilization and comparable pain at rest. These findings suggest that earlier physiotherapist‐supervised mobilization within a structured multimodal perioperative pathway was not accompanied by an increased risk of in‐hospital falls.

Several factors may have contributed to the observed association. Continuous femoral nerve block has been suggested to increase the risk of postoperative falls by impairing quadriceps function. However, evidence from meta‐analyses of randomized controlled trials comparing femoral nerve block with LIA has not demonstrated a consistent difference in postoperative fall rates, with comparable analgesic efficacy [9, 10, 16, 17].

In the present study, LIA was combined with lower opioid consumption and physiotherapist‐supervised mobilization on the day of surgery. Reduced opioid exposure may have limited sedation, dizziness and impaired coordination [8, 12], whereas supervised mobilization may have supported early functional recovery [3, 5, 6, 18, 19]. In contrast, postoperative haemoglobin decline was similar between groups, making reduced blood loss an unlikely principal explanation despite the established importance of perioperative blood management [11]. Taken together, the findings support the concept that postoperative stability may depend on the interaction between motor‐sparing analgesia, reduced opioid exposure and structured mobilization rather than on any single measure.

The 6.6% fall rate in the conventional‐care group was higher than the 2.7% reported by Wasserstein et al. [15]. This difference may reflect variation in event capture rather than a true difference in patient safety. Falls in the present study were prospectively documented through a mandatory institutional incident‐reporting system, which may have captured minor events that could be missed in registry‐based analyses. In addition, the approximately 7‐day inpatient stay provided a longer observation period during which falls could occur and be documented.

Although the number of events was small, their clinical consequences were relevant. Even without fractures, postoperative falls may delay rehabilitation, increase healthcare utilization and reduce confidence during mobilization [12]. The clinical implication is therefore not simply that patients should mobilize earlier, but that early mobilization should occur within a structured pathway incorporating motor‐sparing analgesia, limited opioid exposure and professional supervision. This coordinated approach may support early postoperative safety while preserving the recognized functional benefits of accelerated recovery.

## LIMITATIONS

This study has several limitations. Its retrospective design is susceptible to temporal bias and residual confounding, including unmeasured differences in preoperative mobility, previous falls, neurological conditions, medication use and frailty. The small number of fall events resulted in imprecise effect estimates, as reflected by the wide confidence interval. The pathway was implemented as a bundled intervention, preventing determination of the relative contribution of individual components, and adherence to a comprehensive ERAS Society pathway was not assessed. The single‐centre setting, relatively long inpatient stay and reliance on an institutional reporting system may limit generalizability and introduce reporting bias. Outcomes were primarily restricted to the early inpatient period; longer‐term patient‐reported outcomes, readmissions, late falls and functional recovery were unavailable. The small number of complications also means that secondary safety findings should be considered exploratory. Accordingly, the findings should be interpreted as evidence of an association rather than proof of causality.

## CONCLUSIONS

Implementation of an ERAS‐based perioperative pathway was associated with a lower rate of postoperative in‐hospital falls after primary TKA. These findings support the use of a structured pathway combining motor‐sparing analgesia, reduced opioid exposure and physiotherapist‐supervised early mobilization. Larger prospective studies are required to confirm the observed association.

## AUTHOR CONTRIBUTIONS

Wolf Petersen conceived and designed the study. Judith Ewert collected the clinical data, performed the statistical analysis and drafted the manuscript. Yizhou Ge contributed to data collection. Wolf Petersen and Jan‐Peter Braun critically revised the manuscript. Martin Häner contributed to the interpretation of the data and manuscript revision. All authors read and approved the final manuscript.

## CONFLICT OF INTEREST STATEMENT

The authors declare no conflict of interest.

## ETHICS STATEMENT

The study was approved by the Ethics Committee of the Faculty of Medicine at Charité—Universitätsmedizin Berlin (approval number: EA2/137/20). All patients provided informed consent for the collection of postoperative patient‐reported outcomes and the scientific use of data recorded during the inpatient stay.

## Data Availability

The data that support the findings of this study are available on request from the corresponding author. The data are not publicly available due to privacy or ethical restrictions.
